# Psychological consequences of Covid on general population

**DOI:** 10.1192/j.eurpsy.2023.888

**Published:** 2023-07-19

**Authors:** K. Avramidou, M. Theodoratou

**Affiliations:** 1 Social Sciences, Hellenic Open University, Patras, Greece; 2 Health Sciences, Neapolis University of Pafos, Pafos, Cyprus

## Abstract

**Introduction:**

Pandemics affect the mental health of individuals as they cause specific reactions throughout society due to the measures taken to contain them, which lead individuals to change their behaviour and habits and generally change their daily routine and lifestyle. In addition, the real or perceived threat of the virus and what it may cause exacerbates the anxiety and stress experienced by individuals.

**Objectives:**

To assess the psychological distress experienced by the Greek population due to COVID-19 and to investigate the possible determinants that contributed to negative psychological experience.

**Methods:**

Methodology: A synchronic study was conducted involving 200 participants from different regions of Greece. Data collection took place from October to December 2021. The mean age of the participants was 33.5 years. 76% of the participants had a family and/or friend who had COVID-19, while 27.5% of the participants had a family and/or friend who was hospitalized in the ICU due to coronavirus. The Impact of Event Scale-Revised (IES-R-Gr) questionnaire, adapted for COVID-19, was used to assess mental disorders

**Results:**

40% of participants had no symptoms of PTSD. 14.5% of participants had symptoms of PTSD and 45.5% of participants had a probable diagnosis of PTSD. Women, participants with worse self-assessed health status and participants who had a relative and/or friend hospitalized in ICU due to COVID-19 experienced worse health outcomes due to the pandemic (p<0.001). In addition, women and participants, who had a family and/or friend who was hospitalized in ICU due to COVID-19, were more worried about the pandemic, while participants with worse health status assessment had more nervousness due to the pandemic.

**Conclusions:**

Pandemic is an unprecedented situation experienced by people and has an impact not only on the physical but also on the mental health of the population. Therefore, special attention should be paid to the impact of COVID-19 on the mental health of the population and measures should be taken to protect the mental health of individuals.

**Disclosure of Interest:**

None Declared

